# Association of Plasma Circulatory Markers, *Chlamydia pneumoniae*, and High Sensitive C-Reactive Protein in Coronary Artery Disease Patients of India

**DOI:** 10.1155/2009/561532

**Published:** 2009-04-05

**Authors:** Hem Chandra Jha, Pragya Srivastava, Rakesh Sarkar, Jagdish Prasad, Aruna Singh Mittal

**Affiliations:** ^1^Institute of Pathology (ICMR), Safdarjung Hospital Campus, P.O. Box 4909, New Delhi 110 029, India; ^2^Department of Cardio Thoracic & Vascular Surgery, Safdarjung Hospital, New Delhi 110 029, India

## Abstract

Plasma inflammatory markers have been shown to be predictors for cardiovascular risk, however, there is no study where the levels of plasma circulatory markers have been evaluated in coronary artery disease patients (CAD pts) positive for *C. pneumoniae* IgA and high sensitive C-reactive protein (hsCRP) which may help in better understanding of disease pathogenesis. A total of 192 patients and 192 controls attending the Cardiology Outpatient Department of Safdarjung Hospital were enrolled. The levels of plasma circulatory inflammatory markers were evaluated by ELISA. The levels of circulatory plasma markers (IL-4, IL-8, IL-13, ICAM-1, and VCAM-1) were significantly higher, whereas, levels of IL-10 and IFN-*γ* were significantly lower in CAD pts compared to healthy controls. The levels of IL-4, IL-8, and ICAM-1 (*P* = .007, .015, and .048) were significantly higher, however, IL-10 and IFN-*γ* were significantly lower (*P* < .001, < .001) in *C. pneumoniae* IgA positive CAD pts. The levels of IL-4, IL-8, IL-13, ICAM-1, and VCAM-1 were higher but not significant and levels of IL-10 and IFN-*γ* were significantly (*P* < .001, < .001) lower in hsCRP positive CAD pts. Our study suggested that circulatory cytokines, namely, IL-4, IL-8, and adhesive molecules like ICAM-1 were enhanced after infection with *C. pneumoniae* whereas in contrast to this IL-10 and IFN-*λ* were lowered. Suggesting the important role of these cytokines in progression of CAD.

## 1. Introduction

Atherosclerosis is an inflammatory disease which may be the outcome of responses to microbial antigens [1, 2]. Several conserved components of the bacterial cell wall have been shown to bind to receptors on the cell surface of monocyte and macrophages which may induce production of proinflammatory cytokines [3]. The role of chronic low-grade infection of the arterial wall with *Chlamydia pneumoniae* (*C. pneumoniae*) in the pathogenesis of atherosclerosis has been suggested in a series of epidemiological and pathological studies and may induce innate immunity, molecular mimicry, and autoimmunity as well as direct infection of tissues [4–8]. Earlier prospective studies have shown CRP to be a strong independent predictor of coronary events [1]. It seems that the macrophages deactivating cytokines IL-4 and IL-13 display proinflammatory activities in the vascular system [9] and that IL-8 may have a role as a leukocyte chemoattractant during atherogenesis [10]. Intercellular adhesion molecule-1 (ICAM-1), a major adhesion receptor expressed on the endothelium, is involved in monocyte adhesion to endothelial cells [11]. The vascular adhesion molecule (VCAM-1) has been reported to bind particularly to those classes of leukocytes found in nascent atheroma consisting of the monocyte and the T lymphocyte [12]. Thus, proinflammatory cytokines may link hypercholesterolemia to VCAM-1 expression [13]. We reported earlier association of hsCRP and IL-6 with *C. pneumoniae* IgA serology in CAD patients [14, 15], however, there is no study where levels of these plasma circulatory markers in *C. pneumoniae* IgA positive and hsCRP positive groups of CAD patients have been evaluated which is required for understanding pathogenesis of CAD. Hence the aim of this study was to perceive the levels of plasma circulatory inflammatory markers in CAD patients in the presence of well established CAD markers, namely, *C. pneumoniae* IgA and hsCRP in Indian population.

## 2. Materials and Methods

### 2.1. Patients

A total of 192 patients (148 males and 44 females) attending the Cardiology Outpatient Department of Safdarjung hospital from March 2005 to June 2007 for angiographically confirmed CAD were enrolled for the study after prior written consent. In addition, 192 age and sex matched healthy controls with no evidence of CAD (142 males and 50 females) were also included in the study. The study received clearance from the hospital ethics review committee.

### 2.2. Inclusion Criteria

Evidence of CAD required at least one of the following: (1) significant stenosis (70% of luminal diameter) in at least one major coronary artery proved by angiography and had undertaken either percutaneous coronary intervention or coronary artery bypass graft (CABG); (2) positive stress myocardial perfusion imaging studies for ischemia. 

### 2.3. Exclusion Criteria

Patients were not included if any of the following was present myocardial infarction or CABG in the preceding 3 months, unstable angina, significant valvular heart disease, blood pressure 180/100 mm.

### 2.4. Collection of Samples

Venous blood (5 mL) was collected in nonheparinized tubes from CAD patients and controls. Serum was separated within 2 hours of blood collection and kept at −80°C until used for detection of hsCRP, IL-4, IL-8, IL-10, IL-13, IFN-*λ*, ICAM-1 and VCAM-1, and antibodies against *C. pneumoniae* IgA.

### 2.5. Serology and Antibody Level of Atherosclerotic Marker

Detection of antibodies for *C. pneumoniae* specific IgA was performed using commercially available ELISA kit (R-Biopharm AG, Germany), as mentioned earlier [8]. For detection of antibodies to hsCRP, ELISA was performed using kits (Calibiotech Inc., Calif, USA) as per manufacturer's instructions and level of the hsCRP (>3 mg/L) in serum was considered as hsCRP positive in dichotomized result. Detection of concentration of interleukins (IL-4, IL-8, IL-10, IL-13, and IFN-*λ*), adhesive molecules (ICAM-1 and VCAM-1) was performed using commercially available ELISA kit (e-biosciences, San Diego, Calif, USA) and (Diaclone, France) as, respectively, manufacturer's instructions. Sensitivity and specificity for all kits used in the study were >95%. 

### 2.6. Statistical Analysis

Differences between two groups were evaluated using Mann-Whitney *U*-test. For comparing binary related characteristics *χ*-square test, fisher-exact statistic was used. Simultaneously, an alpha level of 0.05 was set as the level of significance.

## 3. Results

### 3.1. Classification of CAD Patients and Controls on the Basis of Age and Sex

Three age groups of CAD patients and controls were included in this study, however, none of the groups were found to be significantly different. Males and females were also not found to be significantly different when CAD patients and controls were compared (Table 1). 

### 3.2. Evaluation of Plasma Circulatory Markers in CAD pts and Controls

Levels of plasma circulatory markers viz IL-4, IL-8, IL-13, ICAM-1 and VCAM-1 were significantly higher (*P* < .001, <.001, .004, <.001, <.001) in contrast to, levels of IL-10 and IFN-*λ* which were significantly lower (*P* < .001) in CAD pts as compared to controls (Table 2).

### 3.3. Evaluation of Plasma Circulatory Markers in C. Pneumoniae IgA Positive CAD pts and Controls

Mean levels of IL-4, IL-8, and ICAM-1 were significantly higher (*P* = .007, .015, <.001, <.001, and .048) in *C. pneumoniae* IgA positive CAD pts as compared to *C. pneumoniae* IgA negative CAD pts, whereas the levels of IL-10 and IFN-*λ* were significantly lower (*P* < .001, <.001). Mean levels of IL-4 were significantly higher (*P* < .001) and IFN-*λ* was significantly lower (*P* = .006) in *C. pneumoniae* IgA positive controls as compared to *C. pneumoniae* IgA negative controls (Table 3).

### 3.4. Evaluation of Plasma Circulatory Markers in hsCRP Positive CAD pts and Controls

Mean levels of plasma circulatory markers IL-4, IL-8, IL-13, ICAM-1, and VCAM-1 were higher but not significant while levels of IL-10 and IFN-*λ* were significantly lower (*P* < .001) in hsCRP positive CAD pts as compared to hsCRP negative CAD pts. Additionally, similar results were found in hsCRP positive controls as compared to hsCRP negative controls (Table 4).

## 4. Discussion

A variety of plasma inflammatory markers have been shown to predict future cardiovascular risk [16] and is useful for risk stratification and also for identifying those patients who may benefit from targeted interventional therapy. It has been reported that *C. pneumoniae* induces much more IL-6 and IL-8 and lower levels of IFN-*γ* compared to other Gram-positive bacteria [3, 17]. The induction of monocyte procoagulant activity with either IL-6 or IL-8 has been proposed as a possible link between inflammation and thrombosis in patients with CAD [18]. IL-8 has also been reported to play a minor role in mediating monocyte recruitment and adhesion associated with neutrophil chemotaxis [19]. Increased expression of cell adhesion molecules in response to infection with *C. pneumoniae* has also been reported [20, 21]. In our study levels of cytokines IL-4, IL-8, and ICAM-1 were detected higher and levels of IL-10 and IFN-*λ* were lower in *C. pneumoniae* positive compared to *C. pneumoniae* negative CAD patients. Moreover, levels of IL-4, IL-8, IL-13, ICAM-1, and VCAM-1 were detected higher but not significant in hsCRP positive CAD pts compared to hsCRP negative CAD patients. It has been earlier reported that CRP clearly enhances IL-8 production at 8 to 24 hours of incubation, although a considerably less potent than CRP [22, 23]. However, Gabay et al. showed that IL-4 and IL-13 decreased the levels of CRP in primary hepatocytes and hepatoma hepG2 cells [24]. Kieda et al. reported that leukocyte recruitment to the endothelium is mediated by the interaction of adhesion molecule receptors expressed on the surface of endothelium cells for effective host defense against bacteria [25]. Also it is reported that very late antigen-4 (VLA-4), a major adhesion receptor is expressed by T lymphocytes, mediates T-cell adhesion in the microvasculature via an interaction with VCAM-1 on activated endothelial cells [26]. Kawanami et al. showed that CRP induces VCAM-1 gene expression through NF-*κ*B activation in vascular endothelial cells [27], which is parallel to our findings where elevated hsCRP was detected in serum of CAD patients. In our study among different age groups, levels of *C. pneumoniae* IgA were not significantly different (data not shown). Similar results were reported by Masato et al. whereas it is contradictory to that reported by Hahn et al. [28] and Nishimura et al. [29].

## 5. Conclusion

Overall, our study suggested that circulatory cytokines, namely, IL-4, IL-8 and adhesive molecules like ICAM-1 were enhanced in CAD patients infected with *C. pneumoniae* whereas in contrast to this IL-10 and IFN-*λ* were lowered. Additionally, these cytokines were also enhanced in hsCRP positive CAD-pts, suggesting the important role of these cytokines in progression of CAD.

## Figures and Tables

**Table 1:** Characteristics of coronary artery disease patients and controls.

Characteristics	Age groups	Patients (*n* = 192)	Control (*n* = 192)	*P*-value
Age (years)	35–49	67 (34.89)	78 (40.62)	.292
	50–64	93 (48.43)	85 (44.27)	.474
	65–79	32 (16.66)	29 (15.10)	.780

Sex	Male	148 (77.08)	142 (73.95)	.553
	Female	44 (22.91)	50 (26.04)	.553

Figure in parenthesis indicates % positivity.

**Table 2:** Evaluation of plasma circulatory markers in coronary artery disease patients and controls.

Plasma circulatory markers	CAD patients (*n* = 192)	Controls (*n* = 192)	*P*-value
	mean ± SD	mean ± SD	
IL-4 (pg/mL)	1.29 ± 0.17	0.97 ± 0.12	<.001*
IL-8 (pg/mL)	3.27 ± 0.31	2.36 ± 0.22	<.001*
IL-10 (pg/mL)	1.83 ± 0.16	1.95 ± 0.19	<.001*
IL-13 (pg/mL)	4.81 ± 0.28	4.25 ± 0.27	.004*
IFN-*λ* (pg/mL)	1.58 ± 0.14	1.55 ± 0.16	<.001*
ICAM-1 (ng/mL)	14.38 ± 1.16	13.12 ± 1.11	<.001*
VCAM-1 (ng/mL)	73.91 ± 6.71	54.24 ± 4.84	<.001*

CAD: coronary artery disease; *: significant; SD: standard deviation; IL: interleukin; ng: nanogram; pg: pikogram; mL: mililiter; IFN: interferon; ICAM: intercellular adhesion molecule; VCAM: vascular adhesion molecule.

**Table 3:** Evaluation of plasma circulatory markers in *Chlamydia pneumoniae* IgA positive coronary artery disease patients and controls.

Plasma circulatory markers	CAD patients		*P*-value	Controls		*P*-value
	IgA (+ve) (*n* = 155)	IgA (−ve) (*n* = 37)		IgA (+ve) (*n* = 77)	IgA (−ve) (*n* = 115)	
	mean ± SD	mean ± SD		mean ± SD	mean ± SD	
IL-4 (pg/mL)	1.31 ± 0.13	1.17 ± 0.12	.007*	1.06 ± 0.08	0.92 ± 0.08	<.001*
IL-8 (pg/mL)	3.41 ± 0.31	2.76 ± 0.26	.015*	2.46 ± 0.23	2.31 ± 0.21	.444
IL-10 (pg/mL)	1.82 ± 0.11	1.86 ± 0.11	<.001*	1.95 ± 0.14	1.96 ± 0.15	.879
IL-13 (pg/mL)	4.84 ± 0.41	4.70 ± 0.42	.918	4.34 ± 0.36	4.20 ± 0.40	.773
IFN-*λ* (pg/mL)	1.57 ± 0.14	1.65 ± 0.12	<.001*	1.54 ± 0.14	1.56 ± 0.13	.006*
ICAM-1 (ng/mL)	14.54 ± 1.21	13.75 ± 1.24	.048*	13.16 ± 1.10	13.10 ± 1.09	.528
VCAM-1 (ng/mL)	74.33 ± 6.27	72.21 ± 6.15	.454	54.39 ± 4.76	54.10 ± 4.39	.564

CAD: coronary artery disease; *: significant; SD: standard deviation; IL: interleukin; ng: nanogram; pg: pikogram; mL: mililiter; IFN: interferon; ICAM: Intercellular adhesion molecule; VCAM: vascular adhesion molecule; IgA: immunoglobulin A; +ve: positive; −ve: negative.

**Table 4:** Evaluation of plasma circulatory markers in high-sensitive C-reactive protein positive coronary artery disease patients and controls.

Plasma circulatory markers	CAD patients		*P*-value	Controls		*P*-value
	hsCRP (+ve) (*n* = 116)	hsCRP (−ve) (*n* = 76)		hsCRP (+ve) (*n* = 60)	hsCRP (−ve) (*n* = 132)	
	mean ± SD	mean ± SD		mean ± SD	mean ± SD	
IL-4 (pg/mL)	1.31 ± 0.12	1.26 ± 0.11	.719	1.00 ± 0.13	0.96 ± 0.12	.090
IL-8 (pg/mL)	3.32 ± 0.31	3.20 ± 0.26	.573	2.53 ± 0.21	2.29 ± 0.18	.207
IL-10 (pg/mL)	1.82 ± 0.13	1.85 ± 0.14	<.001*	1.94 ± 0.15	1.96 ± 0.24	<.001*
IL-13 (pg/mL)	4.86 ± 0.30	4.74 ± 0.31	.118	4.30 ± 0.33	4.23 ± 0.30	.668
IFN-*λ* (pg/mL)	1.57 ± 0.12	1.59 ± 0.13	<.001*	1.54 ± 0.19	1.56 ± 0.17	<.001*
ICAM-1 (ng/mL)	14.53 ± 1.07	14.16 ± 1.17	.563	13.25 ± 1.13	13.06 ± 1.09	.330
VCAM-1 (ng/mL)	74.20 ± 7.21	73.47 ± 6.91	.658	54.60 ± 4.92	54.10 ± 4.39	.874

CAD: coronary artery disease; *: significant; SD: standard deviation; IL: interleukin; ng: nanogram; pg: pikogram; mL: mililiter; IFN: interferon; ICAM: intercellular adhesion molecule; VCAM: vascular adhesion molecule; hsCRP: high-sensitive C-reactive protein; +ve: positive; −ve: negative.
